# Neuroimaging revolutionizes therapeutic approaches to chronic pain

**DOI:** 10.1186/1744-8069-3-25

**Published:** 2007-09-11

**Authors:** David Borsook, Eric A Moulton, Karl F Schmidt, Lino R Becerra

**Affiliations:** 1 PAIN Group, Brain Imaging Center, McLean Hospital, 115 Mill Street, Belmont, MA 02478, USA; 2 Imaging Consortium for Drug Development, McLean Hospital, 115 Mill Street, Belmont, MA 02478, USA; 3 Program in Neuroscience Department of Psychiatry and Athinoula Martinos Center for Biomedical Engineering, Department of Radiology, Massachusetts Hospital, 149 13th Street, Charlestown, MA, 02129, USA

## Abstract

An understanding of how the brain changes in chronic pain or responds to pharmacological or other therapeutic interventions has been significantly changed as a result of developments in neuroimaging of the CNS. These developments have occurred in 3 domains : (1) *Anatomical Imaging *which has demonstrated changes in brain volume in chronic pain; (2) *Functional Imaging *(fMRI) that has demonstrated an altered state in the brain in chronic pain conditions including back pain, neuropathic pain, and complex regional pain syndromes. In addition the response of the brain to drugs has provided new insights into how these may modify normal and abnormal circuits (phMRI or pharmacological MRI); (3) *Chemical Imaging* (Magnetic Resonance Spectroscopy or MRS) has helped our understanding of measures of chemical changes in chronic pain. Taken together these three domains have already changed the way in which we think of pain – it should now be considered an altered brain state in which there may be altered functional connections or systems and a state that has components of degenerative aspects of the CNS.

## Background

Various technologies have been used in drug discovery and evaluation including combinatorial chemistry, high throughput screening, pharmacogenomics, and proteomics. More recently, brain imaging has provided novel insights into functional, anatomical, and chemical changes in the human condition that allow us to define new approaches that may assist current drug development efforts [1,2].

**Figure 1:**
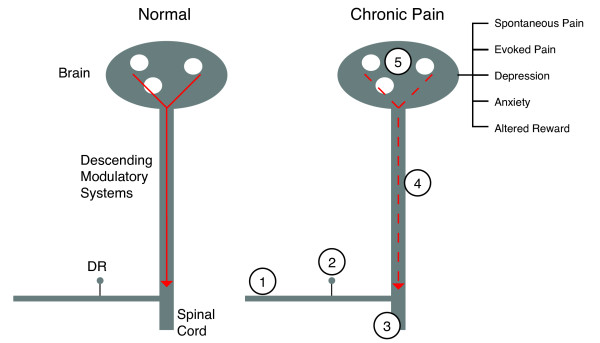
**Summary of Changes in Neural Function following Nerve Injury**. In the Normal State, such as the response to noxious heat (left panel), the pain system provides salient information on the nature of the stimulus, the context of the stimulus, and the emotional response to the stimulus. In the chronic pain state, for example after nerve injury (right panel), changes occur along the neural axis. In the peripheral nerve there may be loss of fibers (1), changes in function of neurons in the dorsal root ganglion (2), and alterations in the dorsal horn (3). In the central nervous system, there are changes in dorsal horn (non-afferent inputs) as well as changes in sensory processing (e.g., hyperalgesia (3 and 5)) and emotional processing (5). These changes also result in an alteration of descending modulatory influences (4) from a number of central nervous system structures (e.g., cingulate, frontal cortex, amygdala etc). The constellation of changes in peripheral and central nervous systems result in changes to the pain intensity during spontaneous and evoked pain, and emotional processing (e.g., altered reward or hedonic state) that may lead to changes in comorbid disease in chronic pain (e.g., depression or anxiety).

A number of findings suggest that our approach to the treatment of chronic pain (e.g., neuropathic pain, fibromyalgia) has been hampered by a lack of understanding of changes that occur subsequent to the onset of the condition at the level of the central nervous system (Figure 1). Amongst these are:

(i) A 'ceiling effect' of current analgesics for chronic pain, rarely exceeding a 30% efficacy level in controlled trials [3] or as defined by numbers needed to treat (NNT) or need to harm (NNH) [4].

(ii) Chronic pain may express itself as a consequence of other conditions. For example, chronic pain may arise after the onset of depression, even in patients without a prior pain history of depression [5].

(iii) Chronic pain patients are defined as 'difficult patients' in that they often have neuropsychological changes that include changes in affect and motivation or changes in cognition, all of which rarely predate their pain condition.

(iv) In some conditions such as complex regional pain syndrome (CRPS), manifestations of dysautonomia, movement disorders, and spreading pain (ipsilateral and contralateral) are all indicative of complex secondary changes in the CNS that follow a relatively trivial peripheral nerve injury [6].

(v) Chronic opioid therapy (e.g., methadone maintenance) results in a hyperalgesic state in both experimental and clinical pain scenarios [7] implying changes in central processing (e.g., alterations in modulatory systems).

(vi) Opioids, arguably the closest approximation to an ideal analgesic, fail to produce pain relief in all individuals, even at high doses. This implies the development of 'analgesic resistance', a consequence of complex changes in neural systems in chronic pain that complicates the utility of opioids for long term therapy [8].

Taken together, these clinical insights suggest a few important points related to pharmacological treatment for chronic pain. First, that pain therapy clearly still requires further research to define a basic understanding of the nature of the disease (particularly regarding CNS processing) that will allow improved analgesic agents in the human condition. Second, that single targets are probably not 'ideal' analgesics given the complexity of the disease affecting the peripheral and central nervous system. Third, a strategy for treating pain is required, given that it is a chronic disease with a dynamic process (e.g., evolution of co-morbid phenotypes such as anxiety or depression) that is not easily reversed in most patients, and alterations in brain anatomy (e.g., cortical thickness) can arise as a consequence of the disease. Recent insights gained from structural and functional imaging the central nervous system (CNS) have provided the groundwork for a novel approach to developing therapies for chronic pain.

**Figure 2:**
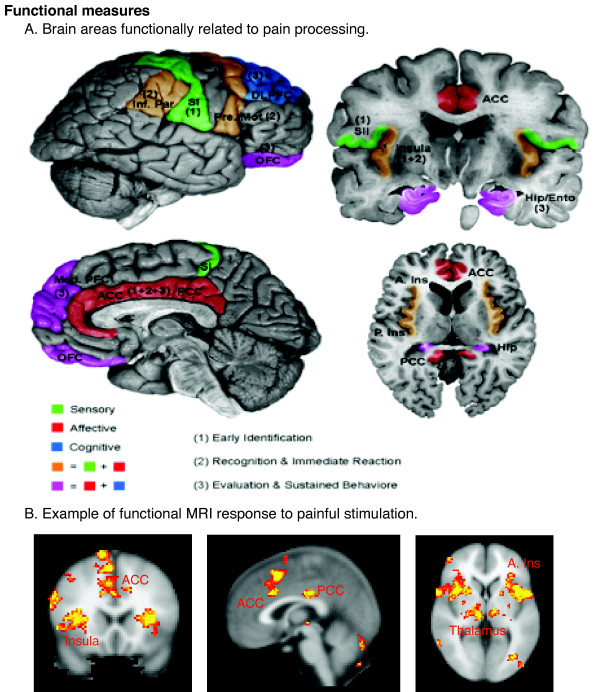
**Examples of CNS Functional Measures**. A. Schematic of cortical areas involved with pain processing. The highlighted areas summarize areas found active in previous functional imaging studies. Color-coding reflects the hypothesized role of each area in processing the different psychological dimensions of pain. Numbers in parentheses indicate the relative involvement of these areas during different temporal stages of the pain experience. Areas displayed include insula, anterior cingulate cortex (ACC), posterior cingulate cortex (PCC), primary somatosensory cortex (SI), secondary somatosensory cortex (SII), inferior parietal lobe (Inf. Par), dorsolateral prefrontal cortex (DLPFC), pre-motor cortex (Pre-Mot), orbitofrontal cortex (OFC), medial prefrontal cortex (Med. PFC), posterior insula (P. Ins), anterior insula (A. Ins), hippocampus (Hip), entorhinal cortex (Ento). [Reprinted with permission from Casey and Tran, 2006]. For examples of brainstem involvement in pain processing, please refer to Tracey and Iannetti ([52]). B. Example of fMRI responses to painful phasic thermal stimulation to the forehead in a cohort of 12 subjects. (Moulton et al., unpublished observations).

**Figure 3:**
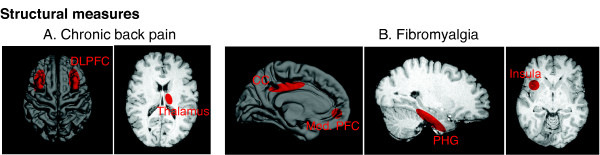
**Schematic Examples of CNS Structural Changes**. Red circles signify decreased gray matter density relative to controls. A. Subjects with chronic back pain show decreases in gray matter density in bilateral dorsolateral prefrontal cortex (DLPFC) and right anterior thalamus (adapted from [25]). B. Patients with fibromyalgia show decreases in cingulate cortex (CC), medial prefrontal cortex (Med. PFC), parahippocampal gyrus (PHG) and insula (adapted from [27]). 3-D surface renderings were created using Freesurfer.

**Figure 4:**
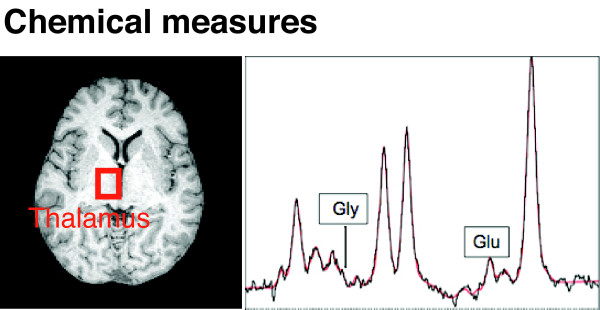
**Example of chemical measures**. The relative concentration of neurotransmitters, such as glutamate (Glu) and glycine (Gly), can be measured using MRS. Here, an *in vivo *proton MRS spectrum focused on the thalamus is displayed in a patient with chronic pain.

Chronic pain is a multidimensional process that now must be considered as a chronic degenerative disease not only affecting sensory and emotional processing, but also producing an altered brain state. Therapeutic interventions should be reconsidered in this context. Several functional (Figure 2), neurochemical, and structural changes have now been defined in the CNS of humans using functional, chemical, and structural (Figure 2, 3, 4) neuroimaging techniques. These changes not only provide novel insights into the pathology of the disease but also opportunities for objective indices for therapeutic efficacy.

### Functional changes

Imaging patients with chronic pain is challenging. Nevertheless, a number of research groups have reported significant changes in pain processing at a functional level including allodynia, functional plasticity, and alterations in basic processes in the brain and brainstem [9-13]. Many of these functional changes have been defined in the context of evoked pain. More recently, basal pain levels have been measured using other approaches, including functional connectivity of how networks are coupled together [14]. Measures of resting state networks have significant implications in terms of understanding and defining the brain state in different chronic pain conditions and the have potential to measure therapeutic efficacy. Such resting state networks have been reported to be consistent across healthy subjects [15] and altered with drug use [16] or disease state [17].

### Neurochemical Changes

Alterations in neurotransmitters have also been reported in chronic pain patients using magnetic resonance spectroscopy (MRS) [18]. Such approaches have been applied to migraine [19], back pain [20,21], and to spinal cord injury [22]. The approach can be used to define neuronal and axonal markers [23], including specific metabolites such as glutamate, aspartate, glycine, and GABA. Furthermore, the use of 19F-NMR as a non-invasive probe allows for measurements of pharmacokinetics of drugs at target sites as well as changes in patient disease state [24]. Neurochemical changes can define biomarkers that precede structural changes.

### Structural Changes

At a macroscopic level, a number of papers have indicated changes in volume in brain regions in patients with chronic neuropathic pain [25], CRPS [26], and fibromyalgia [27]. These last two papers have been seminal in transforming our approach and thinking on chronic pain, since these changes indicate the potential of chronic pain being a neurodegenerative disease. At a microscopic level, changes in dendritic spine density or alterations in neuronal count have been observed in pain and stress [28]. Such changes also have implications for the development of co-morbid disease such as depression [29].

In chronic pain conditions, there is an altered internal milieu as a result of external inputs, altered endogenous processing, or both. Chronic pain resulting from physical (e.g., surgery, trauma) or emotional (abuse, torture, depression) events produce changes in gene function that result in alterations in neural circuits, neural integrity, and receptor function in the CNS. The result is the phenotypic expression of spontaneous pain and increased sensitivity to painful and normally non-painful stimuli (e.g., brush, pressure, thermal). In addition, the condition is sensitive to less obvious perturbations such as changes in barometric pressure, or exacerbated in generalized inflammatory conditions such as the flu.

## Current therapies – What they may tell us

In the past, many drug treatments have focused on the peripheral nerve and dorsal horn. However, most drugs used in chronic pain have CNS effects. These drugs fall into three main categories: opioids, antidepressants, and anticonvulsants. Though not developed for treating pain using a rational mechanistic approach, nearly all of these drugs have been applied to chronic pain treatment. Even in the case of the opioids, subtle effects occur as a result of multiple subtypes of specific receptors (e.g., μ1, μ2 and μ3) or as a result of actions at multiple opioids receptor sites. In the case of antidepressants, the initial mechanism of action on monoamine oxidase (MAO) inhibition has been extended to possible inhibition of sodium channels [30]. For the anticonvulsants, there has always been an association with probable efficacy in chronic pain, dating back to the use of carbamazepine in trigeminal neuralgia [31]. Indeed, the cross domain therapeutic flow has been very fruitful in many cases, of which gabapentin is probably the most well known. Most anticonvulsants have or are also being tried as treatments for neuropathic pain states. While mechanistic approaches (that require careful evaluation of specific responses e.g., cold, heat, von Frey etc) to novel therapies have significant appeal, in practice these may be difficult to implement in busy clinical practice.

In nearly all cases, drugs used in neuropathic pain have actions on the CNS. These actions are not well defined in terms of specificity at the receptor/gene level or at specific regions within the brain. In addition, in most, if not all controlled trials drugs for chronic pain have a ceiling effect in their efficacy of approximately 30%.

## A new focus for therapy

What can we learn from our past? Few analgesics have been designer drugs (e.g., triptans for migraine), and most agents that have come into clinical use by serendipity, or 'parallelism' (e.g., many if not all anticonvulsants have a role in neuropathic pain). Either we are targeting only a small fraction of CNS processes in chronic pain dysfunction or we are not stopping a progressive underlying degenerative process with the current therapies. With respect to the first this may be considered in terms of systems function (e.g., emotional circuitry may be more important than sensory systems), non-neuronal systems involved in CNS function, or neural plasticity that continues to change over time. The latter feeds into the second issue. If chronic pain produces brain matter loss in regions of the brain (e.g., for example the dorsolateral prefrontal cortex) with associated consequences in altered neural function (e.g., cognitive, emotional), then our focus is very different or should be enlarged. In this context, we should now also be evaluating drugs that have better 'neuroprotective effects". The limited information from the use of NMDA inhibitors that are currently available (e.g., ketamine, memantine, methadone – seems to have a combined opioids/NMDA antagonist action) suggests that this may be the issue at hand.

Unusual drugs (i.e., outside of the purview of opioids, anticonvulsants, or antidepressants) provide novel insights into the chronic pain condition. There are a number of drugs candidates for chronic pain therapy that are examples pharmacological agents outside of the 'standard' trio of medications. These include, amongst others, toxins [32], antibiotics. ([33,34], neuroprotective agents [35], glial-modulating agents [36,37], and centrally acting cannabinoids [38]. Many of these have multiple known actions – for example, neurotoxins have anti-convulsant effects [39]; cannabinoid type drugs may have an effect on cytokine modulation ([40], on reward pathways [41], or they may be neuroprotective [42].

Two pharmacological models provide significant insights into the treatment of chronic pain: (i) drug induced changes in central sensitization in an experimental model of pain and (ii) the use of a novel therapy based on neurochemical and neurodegenerative changes in chronic pain.

### Modulation of central sensitization

Central sensitization is observed following experimental tissue injury and in clinical conditions such as inflammation, neuropathic pain, migraine, and perhaps other chronic pain conditions. One fMRI study evaluated the effects of gabapentin on CNS activity evoked during nociceptive mechanical stimulation of a zone of secondary hyperalgesia induced by capsaicin, a model of central sensitization that may parallel some aspects of neuropathic pain [43]. The results indicated that the drug decreased activation in the brainstem during central sensitization, and also suppressed stimulus-induced deactivations with central sensitization. Extrapolations of this type of approach may provide standards for evaluating and comparing drugs in surrogate models of pain.

### Neurochemical protectant

Neuroprotective qualities of a drug can be based on two observations: (i) changes in grey matter volume in the prefrontal cortex and (ii) changes in NMDA concentrations in the frontal regions. The N-methyl d-aspartate (NMDA) antagonist d-cycloserine (also known as the FDA-approved antibiotic, Seromycin) was found to produce a long term decrease in neuropathic pain behavior in rats [34]. The authors argue that the drug may remove memory traces of pain, based on changes in the prefrontal cortex. This type of approach highlights some of the specific insights garnered from neuroimaging.

## The 'perfect' chronic pain drug – Targeting sensory, emotional, and neurodegenerative processes

Current evidence suggests that the perfect pharmacological therapeutic approach would be to: (a) provide early and prolonged pain relief; (b) have peripheral and central effects; (c) have neuroprotective effects; (d) protect against neurodegenerative effects; (d) enhance endogenous analgesic systems though receptor mediated or other mechanisms; and (e) modulate cytokine/immune responses. Such information may now be garnered from neuroimaging approaches potentially helping clinical development programs by enabling the objective evaluation of candidate therapeutics in clinical trials.

Some drugs may actually have a number of these effects in a single agent. Examination of cannabinoid action provides a useful example since it has a number of attributes that make it a useful model as a 'pain drug', as it is: (a) an endogenous mediator (e.g., endogenous receptors); (b) analgesic [44]; (c) a neuroprotectant [45]; and (d) an immune modulator [46,47].

The human condition is not a controlled experimental condition. Unfortunately, many patients seek treatment for pain many months or years after its onset. There are two groups of patients that could benefit from early intervention – diabetics and postoperative patients – who together comprise a significant number of neuropathic pain patients. Treating early with drugs that inhibit or slow down a degenerative process associated with pain may provide long-term effects. While there are patient compliance issues since there may not be an immediate benefit, current evidence suggests that the longer the pain persists, the greater the level of alterations in chronic pain patients.

## Future considerations – Approaches based on new insights

While neuroimaging clearly is not a stand-alone answer to improved pharmacotherapies for chronic pain, recent developments have provided novel insights through studies of the human brain *in vivo *in health and disease. We believe that the application of these insights will help focus therapeutic approaches in clinical trials. Some of these are discussed below.

### Treatment Approaches

Drugs should target three basic processes (a) sensory circuits; (b) emotional circuits; and (c) neural degeneration. Outcome measures would probably be temporally different, with cumulative effects over time. In many ways, the 'Antidepressant Model' may be a useful example, in that the therapeutic effect may only be observed after some time. This may be particularly so with respect to modulation of processes resulting in decreased gray matter volume. For example, the 'Alzheimer Model' has a number of parallels; chronic low dose therapy over months would need to be implemented to achieve stabilization or possible reversal of the condition. The three processes of decreasing pain, enhancing the motivational/emotional status of an individual with chronic pain, and the amelioration of neurodegenerative effects are clearly closely interlinked and benefits in one domain could theoretically affect another. Overall, the ideal intervention would rekindle functional circuits that are directly affected, or would recruit circuits that can take on new functions.

### Multiple Drugs over Time

The notion that one drug may be useful as a single therapy may need to be reconsidered in the light of changes in the nervous system that take place in the chronic pain condition. The rationale for this may relate to considerations beyond 'tachyphylaxis' (i.e., a decreased response with the same repeated doses). including the modulation of genes to induce receptor changes causing them respond differently to a particular drug. This effect has been observed with opioid switching, where the potency of the replacement opioid is greater than if started initially in the patient [48]. These changes can now be evaluated in terms of alterations in specific neural systems using either functional imaging or spectroscopy, and clearly can be applied to different drug interventions including combination therapies.

### Monitoring Progress with Functional, Anatomical or Chemical Scans

Currently there are no objective measures for pain, though recent advances allow for the adoption of specific MRI scans in the clinical domain that may help measure changes in patients. Specifically and perhaps most importantly, such changes may preempt clinical change (e.g., the onset of neuropathic pain following injury). Having said this, there is some evidence in the Alzheimer domain that there may be improvement without any obvious measurable changes using these approaches. Therapies may slow progression or reverse processes that produce anatomical changes. *Functional scans *may be able to define some objective outcomes such as continued central sensitization, responses to specific analgesia (e.g., drug infusion), responses to experimental pain (e.g., thermal or mechanical), or disease process and progress. *Anatomical Scans *may provide information on anisotropy [49] or cortical thickness [50]. *Chemical Scans *can define changes in a number of neurotransmitters as a result of treatment [51].

### Duration of Trials

Most current pain trials last a relatively short time to determine the efficacy of a particular drug. Imaging has changed our understanding that chronic pain is most likely a disease that, if not treated appropriately, progressively alters brain function. Given the nature of chronic pain, data acquired during post-marketing would be useful for assessing the long-term effects of drugs used for chronic pain. The FDA has already implemented some guidelines for post-marketing as a consequence of recent side effect profiles of a number of agents, including some used in pain.

## Conclusion

The ability to evaluate functional, anatomical, and chemical changes in the human brain has provided new insights into the CNS processing of pain in the human condition and also how drugs such as analgesics may work [1]. With respect to the latter, the fact that many analgesics do not provide excellent levels of pain relief indicates that multiple processes are at play in the chronic condition that need to be targeted by new pharmaoctherapies. Some of these targets are unexpected, based on insights garnered from neuroimaging and will almost certainly mould some of our thinking in defining new approaches to discovering analgesics for chronic pain.
